# Bilayer Polycaprolactone (Mitomycin C)/Poly(L-Lactic Acid)/Gelatine (Dimethyloxalylglycine) Nanofibres for Diabetic Wound Repair

**DOI:** 10.3390/membranes16100344

**Published:** 2026-10-16

**Authors:** Renxi Zhu, Qiang Wang, Zhiwei Chen, Feng Chen, Congcong Yang, Jianping Tao, Lunjie Zhao, Yukui Gao, Jingwei Zhang, Yujie Xu, Dong Zhuo, Dan Li

**Affiliations:** 1 Department of Urology, The First Affiliated Hospital of Wannan Medical College, Wuhu 241001, China; 2 Department of Urology, Anhui Provincial Corps Hospital of Chinese People’s Armed Police Force, Hefei 230001, China; 3 Department of General Practice, The First Affiliated Hospital of Wannan Medical College, Wuhu 241001, China

**Keywords:** electrospinning, diabetic wound healing, angiogenesis, anti-fibrosis, dimethyloxalylglycine

## Abstract

**Background**: Diabetic skin wounds arise from chronic hyperglycaemia, peripheral neuropathy, microvascular dysfunction, and compromised immunity. Chronic ulcers are further complicated by infection and local ischaemia–hypoxia, which collectively impede tissue repair. Conventional interventions, including glycaemic control, anti-infective therapy, debridement, and wound dressings, can only partially relieve clinical symptoms. Since these strategies fail to reverse ischaemia–hypoxia or restore the reparative capacity of endogenous cells, ulcers often recur, healing outcomes remain unsatisfactory, and limb amputation may become unavoidable. To overcome these drawbacks, we fabricated a bilayer electrospun microfibre membrane for diabetic wound repair. **Methods**: An electrospun microfibre membrane with a bilayer structure was fabricated. The outer layer, composed of polycaprolactone (PCL), provided mechanical support and enabled sustained release of mitomycin C (MMC). The inner layer, formulated with a 7:3 ratio of poly-L-lactic acid (PLLA) to gelatine (Gel), was designed to match the mechanical properties of the wound bed and was loaded with dimethyloxalylglycine (DMOG). Antibacterial assays against *Escherichia coli* and Staphylococcus aureus, as well as proangiogenic evaluations in ischaemic tissues, were conducted to characterise this composite membrane. **Results**: The outer PCL layer provided mechanical support and enabled sustained MMC release, producing effective antibacterial effects against *E. coli* and *S. aureus*. The DMOG-incorporated inner PLLA/GEL layer displayed favourable mechanical compatibility with wound tissue and successfully promoted angiogenesis under ischaemic conditions. By remodelling the wound microenvironment, the biomaterial improved vascularisation and decreased the bacterial load, ultimately accelerating the repair of diabetic skin wounds. **Conclusions**: This bilayer electrospun membrane integrates antibacterial and pro-angiogenic functions, showing promising application potential for the treatment of diabetic skin wounds.

## 1. Introduction

Skin is the largest barrier of the human body. After an injury, it starts a highly coordinated repair process consisting of haemostasis, inflammation, proliferation, and remodelling [1,2]. This process is controlled by the interaction between keratinocytes, fibroblasts, endothelium cells, immune cells, growth factors, and other parts of the extracellular matrix. In disease-specific diseases such as diabetes, extensive burns, or chronic infections, this process is often disturbed, leading to non-healing chronic wounds, which present a major challenge and a need for advanced regenerative treatment [3]. Successful skin remodelling depends on key cell types such as fibroblasts, macrophages, and endothelial cells to promote the maturation of granulation tissues, build orderly collagen structures, and restore function [4,5]. Traditional treatment of diabetic skin injuries has relied on strict glycaemic control, anti-infective therapy, wound debridement, dressing change, microcirculation and nutrition support, and surgical treatment in certain cases. Recently, skin repair materials have been transformed, moving from passive dressings to bioactive multifunctional solutions capable of actively changing the wound microenvironment, such as electrospun nanofibrous membranes [6], injectable hydrogels [7], controlled growth factor [8] and drug delivery [9], dressings with antibacterial properties [10], and cell-based and 3D-bioprinted skin substitute [11]. Despite these advances, current therapeutic approaches do not address the problem of local ischaemia–hypoxia in diabetic skin wounds and subsequent loss of cell repair. Designing and developing alternative materials that replicate the native structure and function of skin is a promising therapeutic direction.

Electrospinning technology was developed for tissue engineering applications and is now used in various constructions such as skin, blood vessels, and bones. Nanofibres with diameters of 200–600 nm are generated based on the native ECM and provide a structure for cell adhesion and proliferation [12].

Poly(ε-caprolactone) (PCL) and poly(L-lactic acid) (PLLA) are widely used biodegradable polyesters in electrospun wound scaffolds, valued for their processability, mechanical stability, and biocompatibility. PCL offers notable mechanical toughness and degrades slowly, making it well suited for constructing outer protective layers that shield wounds from external irritation and help maintain a stable microenvironment. In contrast, PLLA provides a strong structural matrix for the inner layer; however, its hydrophobic and bioinert nature limits cell adhesion and tissue integration. Gelatine, a hydrophilic biopolymer derived from collagen, is rich in RGD cell-binding motifs. These motifs enhance cell adhesion, spreading, and cytocompatibility, thereby offsetting the hydrophobic and bioinert nature of synthetic poly-L-lactic acid. However, gelatine exhibits weak mechanical strength when wet and degrades rapidly under physiological conditions. As a result, blending gelatine with synthetic polymers is necessary to achieve stable scaffold structures. Dimethyloxalylglycine (DMOG) is a cell-permeable prolyl hydroxylase inhibitor which stabilises HIF-1α under normoxic conditions, upregulates VEGF expression to trigger neovascularisation, and mitigates the ischemic–hypoxic microenvironment of diabetic wounds. Mitomycin C (MMC) is a small-molecule therapeutic agent that exerts broad-spectrum antibacterial activity by blocking bacterial DNA replication. Moreover, MMC suppresses the TGF-β/Smad pathway to alleviate excessive fibrosis and improve wound remodelling quality.

In this study, we demonstrated that combining an outer plasmonic scaffold (PCL/MMC) and an inner membrane (PLLA/GEL/DMOG) produces a bilayer membrane with strong effects on skin repair [13]. The PLLA/GEL/DMOG inner layer exhibits high cell affinity and biological activity. This is largely due to the hydrophobic nature of gelatine, which enhances cell adhesion and proliferation [14,15], while PLLA adds structural stability [15], and dimethyl oxalylglycine stabilises the HIF pathway [16], thereby promoting angiogenesis and reducing ischaemic–hypoxic conditions in chronic wounds, such as diabetic ulcers.

The bilayer membrane offers external protection and targeted internal repair. This dual action stabilises the wound microenvironment and initiates both vascularisation and skin remodelling. As a result, wound healing and functional recovery are significantly improved [17]. The outer PCL/MMC layer is mechanically robust and degrades slowly, providing external support and acting as a physical barrier against external stimuli and tissue collapse [18]. Mitomycin C inhibits bacterial growth by blocking DNA replication during cell division [19], and recent studies indicate that it also suppresses fibrosis by inhibiting the TGF-/Smad pathway [20,21,22].

**Figure 1:**
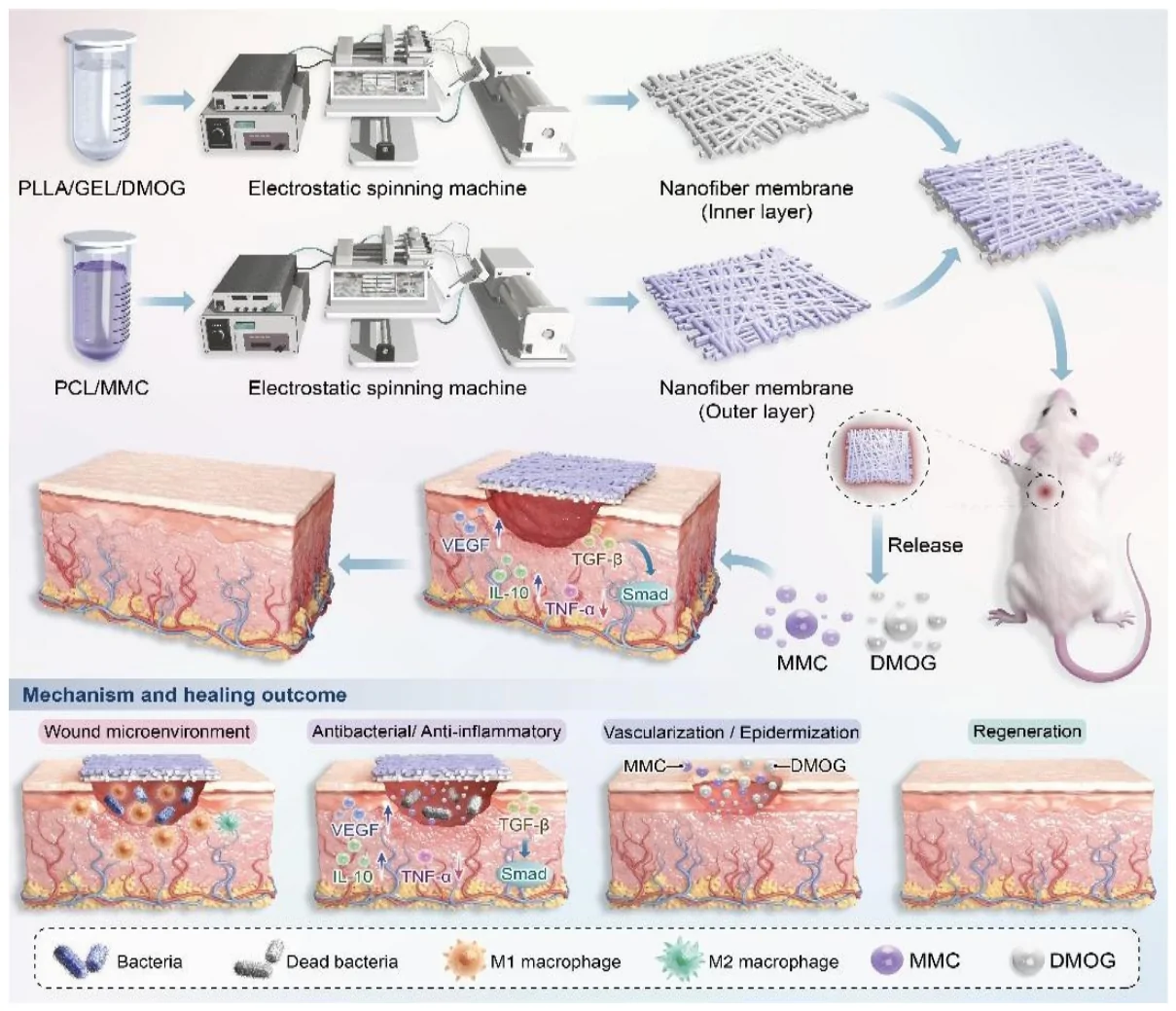
Schematic illustration of the concept and fabrication of the PLLA/GEL/DMOG/PCLMMC electrospinning nanofibre membrane for diabetic wound healing. ↑ indicates an increase, and ↓ indicates a decrease.

Therefore, combining electrospinning technology with PCL, MMC, PLLA, GEL, and DMOG offers a highly promising strategy for developing effective alternative materials for diabetic wounds.The synthesis and application of PLLA/GEL/DMOG/PCL/MMC electrospinning for skin defect repair are shown in Figure 1.

## 2. Materials and Methods

### 2.1. Materials

Poly(L-lactic acid) (PLLA, Mw = 50 kDa), gelatine (Mw = 60 kDa), polycaprolactone (PCL, Mw = 50 kDa), hexafluoroisopropanol (HFIP), mitomycin C (MMC), and dimethyloxalylglycine (DMOG) were obtained from Aladdin (Shanghai, China). Foetal bovine serum (FBS) was supplied by Yisheng (Shanghai, China), while PBS and a streptomycin–penicillin mixture were sourced from Servicebio (Wuhan, China). A 0.25% trypsin solution was purchased from Biosharp (Hefei, China). HaCaT human immortalised keratinocytes, L929 mouse fibroblasts, and their respective culture media were obtained from Yimo Biotech (Shanghai, China). DMEM was sourced from YOBIBIO (Shanghai, China). Calcein/PI cell viability assay kits were procured from Beyotime (Shanghai, China). Goat serum, Alexa Fluor 488 goat anti-rabbit antibody, DAPI staining solution, and primary antibody diluent were purchased from Pinofei Biotechnology (Nanjing, China). Xylene and anhydrous ethanol were obtained from Sinopharm Chemical Reagent Co., Ltd. (Shanghai, China). Primary antibodies against Vimentin, VEGF, IL-10, TNF-α, TGF-β, and Smad2 were purchased from Abways (Shanghai, China).

### 2.2. Preparation of PCL/MMC/PLLA/GEL/DMOG

The PLLA/GEL (7:3) electrospinning solution and the PCL solution were prepared separately by dissolving each polymer in HFIP at a mass fraction of 15%. All electrospinning procedures were conducted at room temperature, with the drum maintained at a rotation speed of 500 rpm. After thorough mixing to ensure a homogeneous PLLA/GEL solution, 8 mL of this solution was transferred into a 10 mL syringe and mounted on the electrospinning apparatus. The process continued under the following conditions: temperature of 20–25 °C, humidity at 30–40%, applied voltage of 15 kV, needle-to-collector distance of 16 cm, and flow (suction) rate of 0.9 mL/h. Once the inner-layer film had been electrospun, 4 mL of the PCL solution was loaded into a 10 mL syringe and processed under comparable conditions, with the humidity level maintained at 30–40%, an applied voltage of 17 kV, a needle-to-collector distance of 12 cm, and a flow (suction) rate of 1.5 mL/h. The resulting PCL/PLLA/GEL bilayer microfibre membrane was then air-dried overnight. For the preparation of drug-loaded membranes, dimethyl oxalamide (DMOG) was incorporated into the PLLA/GEL solution at three concentrations (0.1%/0.2%/0.3%), while 0.1% mitomycin C was added to the PCL solution. These modified solutions were subsequently electrospun under the same operating conditions to obtain PCL/MMC/PLLA/GEL/DMOG membranes.

### 2.3. Morphological Observation of Nanofibre Membranes (SEM)

The nanofibre membranes were examined using a scanning electron microscope (TESCAN MiraLMS, Brno, Czech Republic) operated at an accelerating voltage of 8 kV. Prior to imaging, the sample surface was sputter-coated with a thin gold layer to enhance electrical conductivity and thereby improve image quality. The average diameter of the electrospun microfibres was subsequently determined from the SEM micrographs using ImageJ (1.54f, Bethesda, MD, USA) software.

### 2.4. Water Contact Angle

The wettability of each nanofibre membrane was characterised by measuring the static contact angle using a Dongguan Shengding Precision Instrument SDC-350KS (Dongguan, China). In brief, a droplet of deionised water (0.25 μL) was deposited onto the surface of the nanofibre membrane and allowed to stabilise for 2 s, after which a static image was recorded. The contact angle was then determined directly by measuring the angle formed between the tangent line at the liquid–solid interface and the phase boundary.

### 2.5. Fourier Transform Infrared Spectroscopy (FTIR)

The principal alterations in the chemical composition of the polymer blend were characterised using FTIR (Thermo Fisher Scientific Nicolet iS20, Waltham, MA, USA). Spectra were recorded for finely powdered samples over the range of 4000–400 cm^−1^.

### 2.6. Mechanical Properties

The mechanical performance of the 5 cm × 1 cm nanofibre membranes was evaluated using a universal electronic testing machine (Instron 3367, Norwood, MA, USA) fitted with a 100 N load cell and a fixed gauge length of 1 cm. During the uniaxial tensile testing at a constant crosshead speed of 2 mm/min, tensile stress, fracture strain, and Young’s modulus were continuously recorded up to the point of failure (*n* = 3).

### 2.7. Drug Release and Degradation Experiments

The in vitro release rate of DMOG was quantified via high-performance liquid chromatography (HPLC). The biomaterials were each loaded into centrifuge tubes containing 20 mL of PBS solution at three different concentrations and immediately placed in a constant-temperature shaker set to 100 rpm at 37 °C. At predetermined time points, 1 mL of the supernatant was withdrawn from each tube and replaced with an equal volume of fresh PBS solution to maintain sink conditions. The detection wavelengths were set at 234 nm for DMOG and 365 nm for MMC. All release experiments were performed in triplicate at each time point, and the cumulative drug release percentages were calculated via the external standard method. The 0.1% PGD/PM DMOG group exhibited a loading efficiency of 32.31 ± 1.18%, with an average loaded dose of 64.82 ± 2.36 μg per membrane. The 0.2%PGD/PM DMOG group showed a loading efficiency of 32.45 ± 1.22%, with an average loaded dose of 129.65 ± 4.72 μg per membrane. The 0.3%PGD/PM DMOG group achieved a loading efficiency of 32.49 ± 1.28%, with an average loaded dose of 194.47 ± 7.10 μg per membrane. The outer PCL layer was loaded with a fixed amount of MMC, presenting a loading efficiency of 28.62 ± 1.09% and an average drug loading of 18.72 ± 0.63 μg per membrane.

DMOG degradation was assessed using a repeated mixing protocol. The biomaterials at three different concentrations were suspended in PBS solution (pH 7.4) at 37 °C under continuous stirring for one week; afterwards, the samples were transferred to a 60 °C oven and incubated for one day prior to weighing. This sequence of stirring and 60 °C thermal treatment was repeated for a total of six groups over the seven-week experimental period. The mass loss percentage was calculated as follows:

$$\mathrm{Massloss}\;(\%)=\frac{W_{0}-W_{T}}{W_{0}}$$

where W_0_ is the initial dry weight of the membrane, and W_T_ is the dry weight of the residual membrane after drying at week t.

### 2.8. Antibacterial Test

The antibacterial activity of the bilayer membranes was evaluated via a contact-killing assay. *Staphylococcus aureus* (*S. aureus*) and *Escherichia coli* (*E. coli*) were selected as representative bacterial strains to assess the antibacterial performance of the electrospun nanofibre membranes. The *S. aureus* and *E. coli* bacterial suspensions were adjusted with sterile PBS to a final concentration of (10^5^) CFU/mL, and 100 μL of each suspension was uniformly spread onto Luria–Bertani solid agar. The PLLA/GEL/PCL and PLLA/GEL/DMOG/PCL/MMC specimens were cut into circular pieces with a diameter of 10 mm and sterilised by means of ultraviolet irradiation. The sterilised membranes were then carefully positioned on the agar surface using sterile forceps to ensure complete contact. All antibacterial tests were performed in triplicate biological replicates (*n* = 3). The inoculated plates were incubated at 37 °C for 24 h. After co-culture, the bacteria were eluted with sterile PBS, followed by serial dilution and spread-plating (0.1 mL bacterial suspension per agar plate). The recovered bacterial concentration (CFU/mL) was calculated as follows:

C(CFU/mL) = Colony count × dilution factor × 10 × 100

where the factor of 10 originates from spreading 0.1 mL bacterial suspension on the agar plate, and the factor of 100 corresponds to a 100-fold dilution using sterile PBS for eluting the co-cultured bacteria.

The antibacterial ratio was calculated using the following formula:

$$\mathrm{Antibacterial}\;\mathrm{ratio}\;(\%)\;=\;(1-\frac{C_{\mathrm{sample}}}{C_{\mathrm{control}}})\times 100\%$$

where C_sample_ = bacterial concentration of the experimental group (CFU/mL), and C_control_ = bacterial concentration of the blank control group (CFU/mL).

### 2.9. Cell Compatibility Experiment

Fresh tissues were sampled within 1–3 min, with a tissue block area ≤ 3 mm^2^. After washing with PBS, the samples were fixed in electron microscopy fixative for 2 h at room temperature and stored at 4 °C. Adherent cells were fixed similarly after discarding the medium and washing. The samples were rinsed with PB, post-fixed with osmium acid, dehydrated in graded ethanol, replaced with isopentyl acetate, dried by critical point drying, and sputter-coated with gold. HaCaT and L929 cells were plated and treated for 24 h, followed by CCK-8 assay to measure the absorbance at 450 nm for cell viability assessment.

A Calcein AM/PI staining working solution was prepared, mixed with washed cells, and incubated at 37 °C in the dark for 20 min. The cells were washed with PBS to remove unbound dye and then observed and imaged under a fluorescence microscope; live cells showed green fluorescence, whereas dead cells showed red fluorescence.

All scratch assay tools were sterilised; the rulers and markers were UV-treated for 30 min, and 6-well plates were seeded with logarithmic-phase cells. After confluence, scratches were created. Following PBS washing and serum-free culture, images were acquired at set time points to calculate the cell migration rates.

Logarithmic-phase L929 and HaCaT cells were seeded in 6-well plates and cultured overnight. After 24 h of treatment, the cell slides were fixed, permeabilised, blocked, and incubated with primary and secondary antibodies. DAPI was used to stain the nuclei. The slides were mounted with an anti-fade reagent and stored at 4 °C in the dark.

### 2.10. Animal Experiment

The animal experiment was conducted in strict accordance with the “China National Guidelines for the Care and Use of Experimental Animals”. All experimental procedures and handling protocols were reviewed and approved by the Institutional Animal Care and Use Committee of Wannan Medical University (Approval No.: WNMC-AWE-2025231, 6 March 2025). A diabetic mice model was generated by combining a high-fat diet with low-dose STZ. The experimental animals were fed a high-fat diet for 4 weeks, and STZ was subsequently delivered via intraperitoneal injection at 40 mg/kg. Diabetes was confirmed when the fasting blood glucose level was ≥11.1 mmol/L at 72 h after STZ administration. The average fasting blood glucose level of the diabetic animals was 16.6 ± 2.1 mmol/L. The animals were randomly assigned into three groups using a random number table. The investigators were not blinded during wound measurement and histological analysis. No postoperative analgesia was provided. Prophylactic antibiotics were used after wound surgery to prevent infection, and no splinting system was applied to the wounds. All animals received normal feeding until the predefined experimental endpoint, and no euthanasia was performed in this study. Following model establishment, the mouse were maintained on a standard diet with ad libitum access to water in a controlled environment with a temperature of 25 °C under a 12 h light–dark cycle. A total of 24 healthy mouse were randomly allocated to one of three groups (8 mouse per group): the tissue-engineered PCL/PLLA/GEL group, the 0.2% PCL/MMC/PLLA/GEL/DMOG group, and the blank control group. Full-thickness skin excisions were performed on the dorsal skin to create a 10 mm × 10 mm defect model. No special template or instrument was used for wound creation. The tissue excision penetrated the dermis down to the subcutaneous fat layer. These defects were then repaired using the prepared PCL/PLLA/GEL and PCL/MMC/PLLA/GEL/DMOG microfibre membranes. The wound sites were photographed (digital camera) on days 0, 3, 6, 9, and 12 to monitor the healing process. Following the surgery, all animals were housed individually in separate cages, with unrestricted access to food and water. The wound closure percentage was calculated as follows:

$$Wound\;closure\;(\%)=\frac{A0-At}{A0}\times 100\%$$

where A0 is the initial wound area and At s the wound area at the indicated time point.

Linear regression analysis was performed on the wound healing rate versus time data over the 12-day observation period for each group, using the following equation:

*y* = *ax* + *b*

where y represents the wound healing rate, x is the time (days), the slope a denotes the daily increment of wound healing rate, and b is the intercept. The coefficient of determination (R^2^) was calculated to evaluate the goodness of fit. The slopes (a) of the fitted equations for the three groups were compared to quantitatively assess differences in wound healing rates.

### 2.11. Histological and Immunohistochemical Analysis

Healed skin tissues were harvested on day 12. The *specimens* were immediately fixed in 10% polyformaldehyde for 48 h, after which they underwent stepwise washing, graded dehydration, paraffin embedding, and microtomy to obtain 5 μm sections. Tissue morphology was evaluated using haematoxylin–eosin (HE) staining. Collagen deposition was subsequently assessed at 100× magnification after Masson trichrome, Sirius red, and carmine red staining, and representative images were captured for documentation. In parallel, immunohistochemical (IHC) staining was performed to determine the expression of IL-10, P-AKT, TGF-β, Smad2, TNF-α, and VEGF in the skin tissues.

### 2.12. Statistical Analysis

Data are expressed as mean ± standard deviation (Mean ± SD). Statistical analysis was performed using Analysis of Variance (ANOVA) in the GraphPad Prism software (Version 10.1.2). A *p*-value < 0.05 was considered statistically significant.

## 3. Results and Discussion

### 3.1. Morphology and Diameters of Nanofibre Membranes

**Figure 2:**
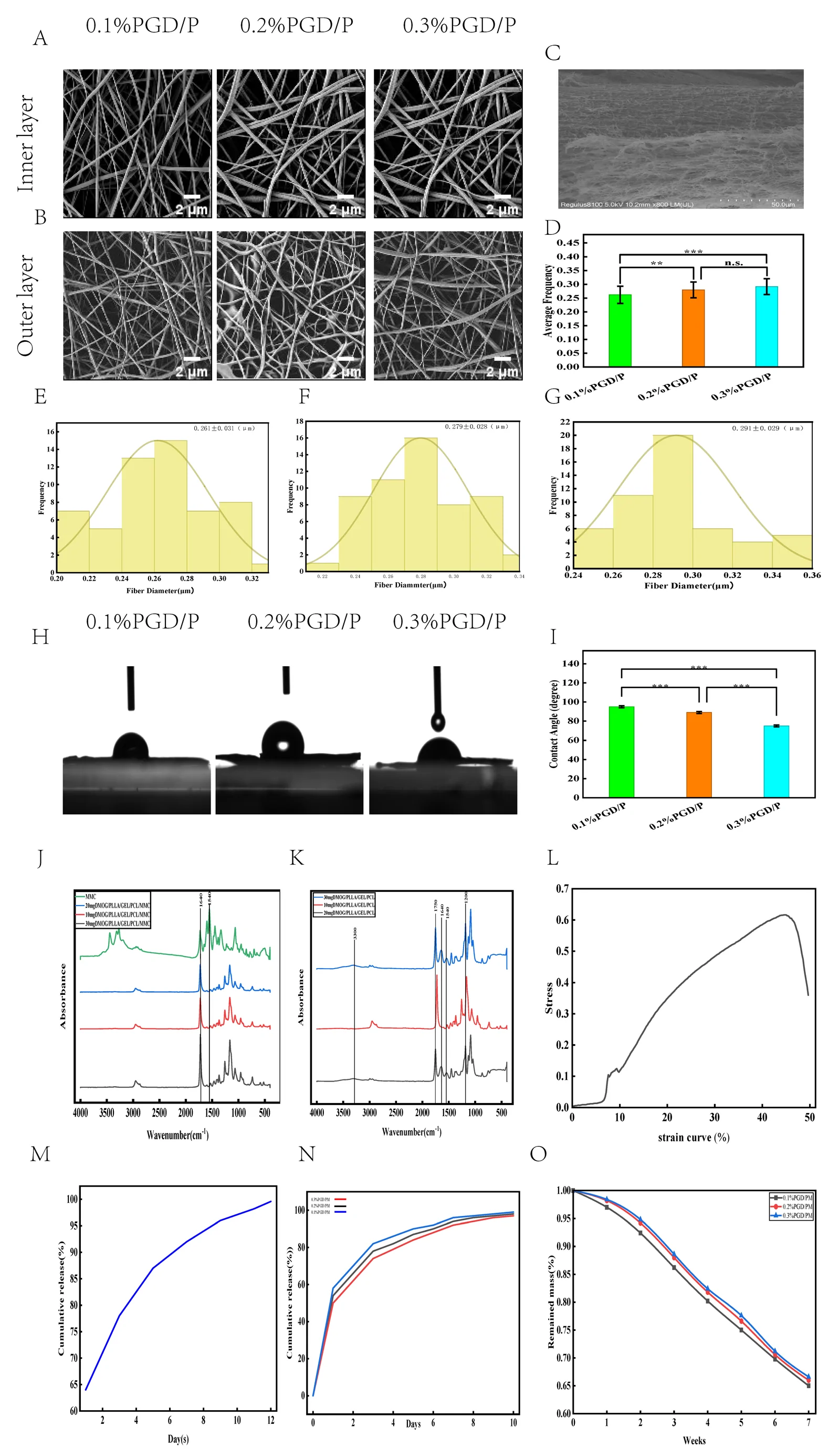
Morphology and physicochemical properties of the PGD/P bilayer nanofibre membranes. SEM micrographs of the inner (**A**) and outer (**B**) layers of the 0.1%, 0.2%, and 0.3% PGD/P membranes (scale bar = 2 μm). (**C**) cross-sectional SEM images. (**E**–**G**) Inner-layer fibre diameter histograms with average diameters of 0.261 ± 0.031 μm, 0.279 ± 0.028 μm, and 0.291 ± 0.029 μm, respectively. (**D**) Average fibre frequency. (**H**) Representative images of water contact angles and (**I**) quantitative results showing contact angles of 95.599°, 89.993°, and 77.367°. (**J**,**K**) FTIR spectra of PGD/P nanofibres. Mechanical characterisation: stress–strain curve (**L**); the membrane possessed a maximum force of 2.51 N, an elongation at break of 49.67%, a tensile strength of 0.62 MPa, a displacement at maximum force of 6.68 mm, and an elastic modulus of 1.76 MPa. (**M**,**N**) In vitro MMC and DMOG release profile and (**O**) material degradation behaviour. ** *p* < 0.01, *** *p* < 0.001, and ns: no significance.

Electrospun nanofibre membranes establish a supportive microenvironment for cell adhesion, growth, and proliferation, which is indispensable for efficient tissue regeneration during skin defect repair [23]. As illustrated in Figure 2A–C, the scanning electron microscopy (SEM) demonstrated that the PCL/PLLA/GEL/DMOG (0.1%, 0.2%, 0.3%) electrospun nanofibre membranes possessed randomly oriented yet structurally homogeneous networks, characterised by continuous, crack-free architectures.

Quantitative assessment of fibre morphology revealed that the average fibre diameters of the three membranes containing different DMOG concentrations at 0.1%, 0.2%, and 0.3% were 0.261 ± 0.031 μm, 0.279 ± 0.028 μm, and 0.291 ± 0.029 μm, respectively, as shown in Figure 2D–G This dimensional stability is largely attributed to the presence of gelatine, which acts as an efficient protective colloid, suppressing crystal aggregation and thereby preserving the structural fidelity of the microfibres [24]. Following DMOG incorporation, the fibres displayed a narrower diameter distribution and a smoother, flatter surface topography, accompanied by a pronounced reduction in bead-like imperfections and fracture defects.

### 3.2. Hydrophilic Analysis of Nanofibre Membranes

As shown in Figure 2H,I, the functionalised PLLA microfibre membrane behaves as a distinctly hydrophilic substrate. Previous work has demonstrated that the incorporation of gelatine increases the surface contact angle, while the abundance of hydroxyl and other polar hydrophilic moieties in gelatine simultaneously augments the overall hydrophilicity of the composite [25]. In agreement with these observations, the GEL-functionalised PLLA microfibre membranes in this study display pronounced hydrophilic characteristics. Such hydrophilic composite scaffolds are known to support cell adhesion and proliferation, while also providing a favourable microenvironment for the diffusion of nutrients and metabolites [26]. The measured water contact angles for the PCL/PLLA/GEL/DMOG (0.1%, 0.2%, 0.3%) nanofibre membranes were 95.599°, 89.993°, and 77.367°, respectively, indicating a progressive increase in surface wettability. Upon loading DMOG onto a PLLA/GEL fibre membrane, the water contact angle is further reduced, thereby enhancing water uptake. As illustrated in the figure, increasing DMOG concentration leads to a monotonic decrease in the water contact angle, which can be attributed to the high density of hydrophilic functional groups in DMOG that reinforces the hydrophilicity of the PLLA/GEL microfibre membrane [27].

### 3.3. FTIR Analysis

In Figure 2J, MMC also exhibits characteristic absorption bands, with carbonyl (C=O) stretching vibrations at 1630–1640 cm^−1^ and additional vibration absorption bands corresponding to nitrogen-containing heterocycles at 1520–1540 cm^−13^ [3]. After incorporating MMC into the nanofibre matrix, no new independent MMC characteristic peaks were observed in the spectrum of the PLLA/GEL/DMOG/PCL/MMC scaffold. The MMC characteristic bands at 1630–1640 cm^−1^ and 1520–1540 cm^−1^ were masked by the strong amide I and amide II absorption of GEL and DMOG. Only a slight change in absorbance intensity at 1520–1540 cm^−1^ could be detected, confirming that MMC was physically encapsulated into the nanofibres rather than chemically bonded to the polymer matrix.

As shown in Figure 2K, FTIR spectroscopy was used to characterise the distinctive absorption bands of the PLLA/GEL/DMOG/PCL and PLLA/GEL/DMOG/PCL/MMC scaffolds. These bands were accompanied by C–H bending at 1450–1460 cm^−1^ and single-bond stretching bands in the 1000–1200 cm^−1^ region, associated with C–O–C linkages in PLLA and C–O/C–N bonds in DMOG. When compared with previously reported FTIR spectra of individual components, the peak positions of our composite membranes were generally consistent with the literature values [3,28,29]. The broad O–H/N–H band at ~3300 cm^−1^ was slightly broadened and shifted to a lower wavenumber relative to pure gelatine or DMOG spectra reported in the literature, which originated from the intermolecular hydrogen bonding formed between hydroxyl and amide groups across PLLA, GEL, PCL, and DMOG within the electrospun nanofibres. The strong ester C=O signal near 1750 cm^−1^ showed a minor reduction in peak intensity compared with neat PLLA/PCL, likely due to the overlapping absorption of DMOG carboxyl groups and partial hydrogen-bonding-induced restriction of carbonyl stretching. The amide I (~1640 cm^−1^) and amide II (~1540 cm^−1^) bands matched well with published gelatine spectra, yet subtle peak broadening was observed as a result of superposition with DMOG amide vibrations. The C–H bending (1450–1460 cm^−1^) and C–O–C/C–O/C–N stretching signals (1000–1200 cm^−1^) were consistent with the fingerprint region of PLLA and PCL reported in prior studies, with only negligible wavenumber variation caused by the blending of multi-components.

### 3.4. Analysis of Mechanical Properties

Figure 2L present the force–deformation profile of the PLLA/GEL/PCL composite scaffold [28]. The mechanical properties of the as-prepared material were evaluated using tensile testing. The tensile strength, elongation at break, and elastic modulus were measured to be 0.62 MPa, 49.67%, and 1.76 MPa, respectively. The force–deformation curve further verified its flexible nature, as the material endured substantial deformation prior to fracture. Proper mechanical matching between wound dressings and native skin plays a vital role in cutaneous wound healing. Benefiting from its low elastic modulus, the material displays desirable softness and can closely conform to irregular wound surfaces. Its moderate stretch capacity allows synchronous deformation with skin during daily movements, mitigating detrimental shear and tensile stress applied to delicate granulation tissues and preventing secondary wound tearing. This favourable mechanical microenvironment facilitates fibroblast migration and collagen deposition, thus accelerating skin regeneration. Nevertheless, its limited extensibility fails to satisfy the large deformation requirements of joint movements, indicating that the dressing is more applicable to wounds on less-mobile skin regions instead of highly flexible joint sites.

### 3.5. Drug Release and Degradation Experiment

As depicted in Figure 2M, MMC was released rapidly on the first day in the PCL/MMC microfibre membrane, with an in vitro cumulative release rate of 63.1 ± 1.6%. From the second day onwards, MMC started to be released continuously and slowly until the 12th day. At this point, the in vivo cumulative release rate reached 99.42 ± 0.03%, indicating that MMC had been basically completely released.

As depicted in Figure 2N, the in vitro release behaviour of DMOG from the PLLA/GEL/DMOG composite was assessed at three loading concentrations (0.1%, 0.2%, and 0.3%). All groups demonstrated a pronounced initial burst, with more than 50% of DMOG released within day 1; afterwards, DMOG release proceeded in a sustained manner up to day 7, yielding cumulative release values exceeding 90% and approaching complete release by approximately day 10. Such a release profile is consistent with the provision of prolonged antibacterial activity and sustained angiogenic stimulation during skin repair [30]. Figure 2O depicts the in vitro degradation of the PGD/PM materials over a period of 7 weeks. Across all groups, degradation followed a gradual and continuous pattern; however, a lower PGD content was associated with more rapid mass loss such that the 0.1% group degraded the fastest and the 0.3% group the slowest, particularly during the first 5 weeks. After week 5, the degradation rate decelerated markedly, and all groups approached a quasi-stable degradation state by week 7. This controlled degradation behaviour, which is attributable to improved structural integrity at a higher PGD content, mitigates local inflammatory responses linked to abrupt release of degradation products, thereby supporting the favourable biosafety of these materials for skin repair applications.

### 3.6. Antibacterial Test

**Figure 3:**
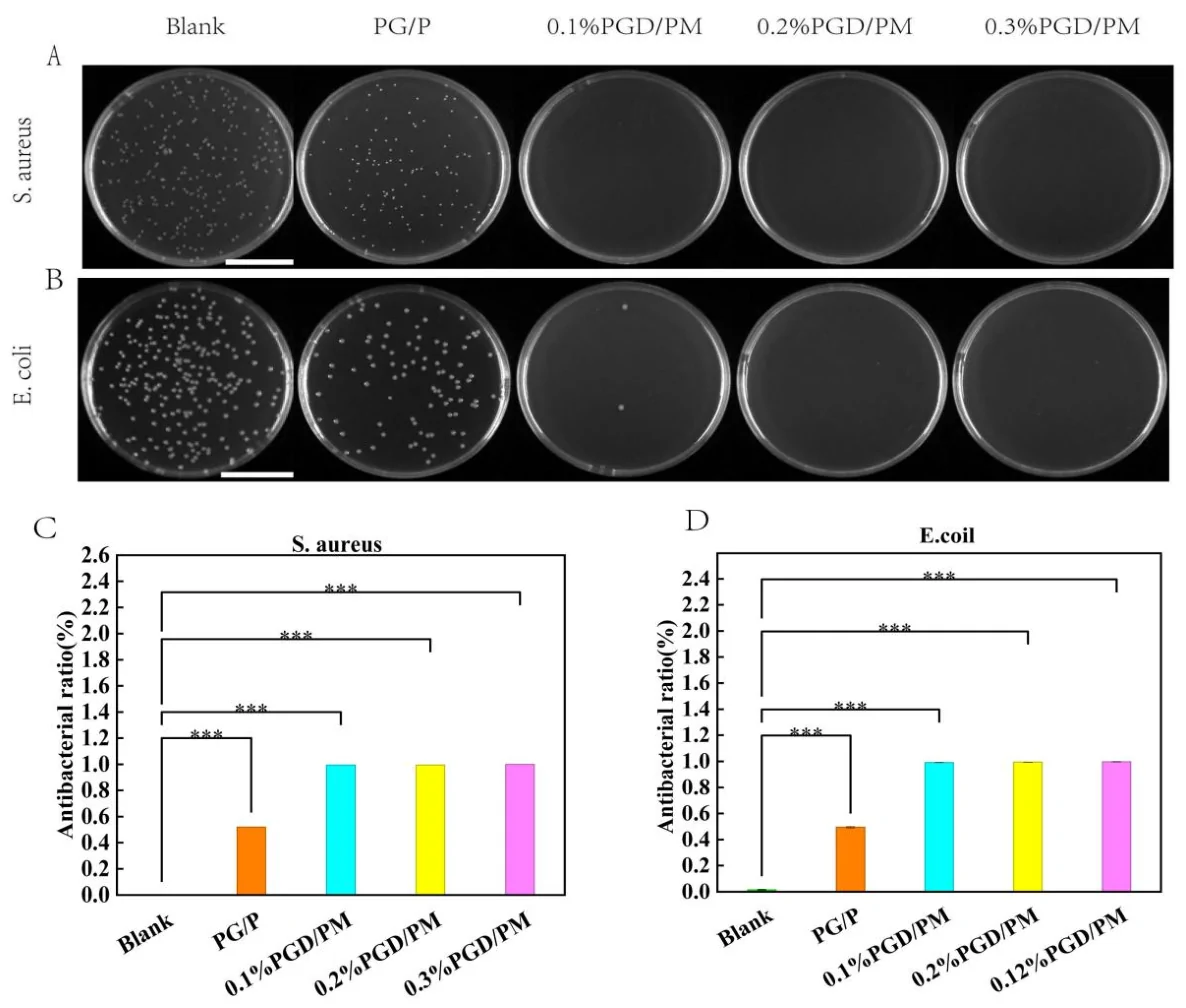
Antibacterial evaluation of the blank, PG/P, 0.1%PGD/PM, 0.2%PGD/PM, and 0.3%PGD/PM groups against *S. aureus* and *E. coli*. Representative colony photographs of *S. aureus* (**A**) and *E. coli* (**B**) (scale bar = 1 cm). Quantitative analysis of antibacterial ratio against *S. aureus* (**C**) and *E. coli* (**D**). *** *p* < 0.001.

Some studies have indicated that MMC interferes with the synthesis of bacterial DNA, which prevents the bacterial genetic material from replicating itself and thus inhibits the growth and proliferation of bacteria [19]. Moreover, MMC compromises the integrity of the bacterial cell membrane, perturbs membrane permeability, promotes the leakage of intracellular constituents and, ultimately, leads to bacterial death [20,31]. As the antibacterial performance of the electrospun nanofibre membrane developed in this study is predominantly attributable to MMC, a randomised combination of the nanofibre membrane and its matrix was selected for assessment. Two representative bacterial strains—*Staphylococcus aureus* (*S. aureus*), shown in Figure 3A,C and *Escherichia coli* (*E. coli*), shown in Figure 3B,D—were employed to compare the antimicrobial efficacy of PG/P and 0.2% PGD/PM, using the bacterial inhibition rate as the primary outcome measure.

Compared with the PG/P group, the 0.1%, 0.2%, and 0.3%PGD/PM groups exhibited substantially higher inhibition rates against both *S. aureus* and *E. coli*. The differences between the groups were statistically significant (***), indicating that PGD/PM provides potent broad-spectrum antibacterial activity and effectively suppresses the proliferation of both Gram-positive and Gram-negative bacteria.

### 3.7. Biocompatibility of Nanofibre Membranes

**Figure 4:**
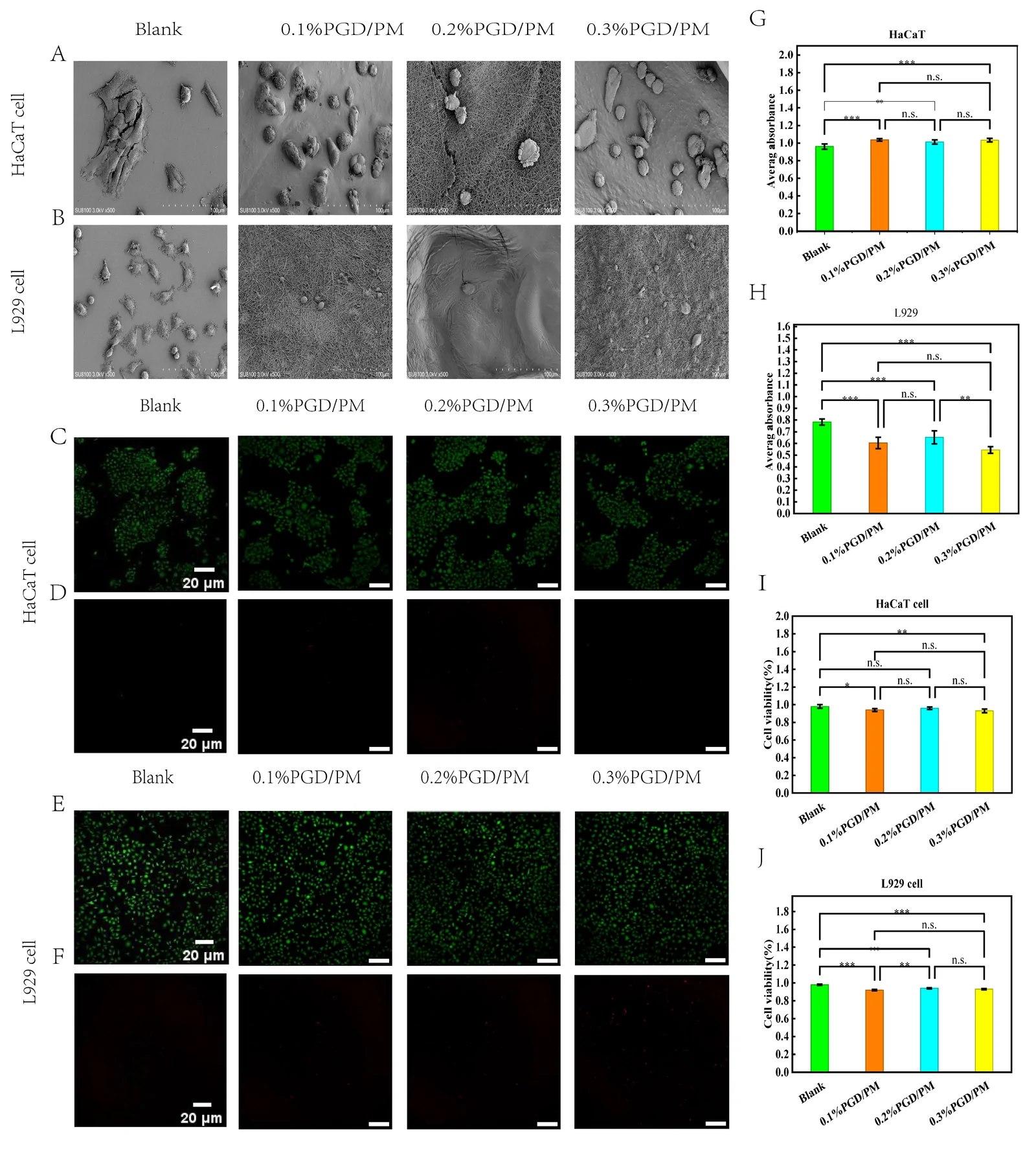
Cellular adhesion, morphology, and biocompatibility of nanofibre membranes. SEM micrographs of HaCaT (**A**) and L929 (**B**) cells cultured on PGD/P nanofibre membranes for 72 h (scale bar = 100 μm). Immunofluorescence staining images of HaCaT (**C**,**D**) and L929 (**E**,**F**) cells cultured on the membranes (scale bar = 20 μm). CCK-8 assay for cell proliferation and viability. The average absorbance values (**G**) for HaCaT cells were 111.11%, 107.62%, and 110.56%, and the average absorbance values (**H**) for L929 cells were 64.20%, 73.77%, and 51.99% across the three groups. Quantitative cell viability for HaCaT cells (**I**) was 92%, 94%, and 93%, while that for L929 cells (**J**) was 94%, 96%, and 93%. * *p* < 0.05, ** *p* < 0.01, *** *p* < 0.001, and ns, no significance.

The biocompatibility of the PLLA/GEL/DMOG/PCL/MMC nanofibre membranes at three concentrations (0.1%, 0.2%, 0.3%) was evaluated through co-culturing with human keratinocytes (HaCaT) and mouse fibroblasts (L929), and cell proliferation was quantified using the CCK-8 assay [32]. As shown in Figure 4G, HaCaT cells in all three groups exhibited cell viability greater than 100%, with the 0.1% group reaching a value of 111.11% (an 11.11% increase vs. the blank control), followed by the 0.3% (110.56%) and 0.2% (107.62%) groups, which indicates robust keratinocyte proliferation. By contrast, Figure 4H shows that L929 cells in all three groups displayed cell viability below 100%: the cell viability of the 0.3% group was lowest, at 51.99% (a 48.01% decrease), whereas the values of the 0.1% and 0.2% groups were 64.20% and 73.77%, respectively, suggesting dose-dependent cytotoxicity toward fibroblasts. Nevertheless, both cell types retained survival and proliferative capacity on the electrospun nanofibre membranes. The SEM analysis (Figure 4A,B) further demonstrated that cells adhering to the membrane surfaces preserved normal morphology, and HaCaT cells displayed a well-spread morphology during proliferation and attachment.

### 3.8. Live/Dead Cell Staining Assay

HaCaT and L929 cells, which represent epidermal and dermal core cell populations, respectively, are commonly used to assess the biocompatibility and skin toxicity of skin implant materials [33]. Live/dead staining, shown in Figure 4C–F, provides direct visualisation of cell survival and allows quantification of mortality following exposure to different material concentrations. As shown in Figure 4I, HaCaT cells in the control group exhibited a survival rate of 98%, whereas cells in the 0.1%, 0.2%, and 0.3% PLLA/GEL/DMOG/PCL/MMC groups showed survival rates of 94%, 96%, and 93%, respectively, corresponding to decreases of less than 10% relative to the control; the survival rates of cells in all groups remained above 90% and displayed no statistically significant differences. Likewise, Figure 4J indicates that L929 cells in the control group had a survival rate of 98%, while cells in the treatment groups showed survival rates of 92% (0.1%), 94% (0.2%), and 93% (0.3%), representing reductions of 6%, 4%, and 5%, respectively. Collectively, these data indicate that none of the three material concentrations induces significant acute cytotoxicity or skin irritation in either epidermal HaCaT keratinocytes or dermal L929 fibroblasts. Notably, CCK-8 metabolic activity and membrane-integrity-based live/dead staining yielded divergent outcomes across the three tested concentrations (0.1%, 0.2%, and 0.3%) [34]. Live/dead staining detects terminal cell death via irreversible plasma membrane rupture and cannot identify sublethal stress without membrane damage, whereas the CCK-8 assay measures mitochondrial dehydrogenase activity, which sensitively reflects early metabolic dysfunction [35]. For HaCaT keratinocytes, both assays showed favourable cytocompatibility relative to the blank control at all concentrations, supporting re-epithelialisation. For L929 fibroblasts, the discrepancy was evident across concentrations and was most prominent for the 0.3% formulation: in particular, the live/dead staining indicated >90% cell survival and intact membranes relative to the blank control, excluding overt cell lysis, while the CCK-8 assay demonstrated markedly suppressed metabolic activity. These findings indicate that the material induces sublethal mitochondrial impairment in L929 cells without irreversible membrane breakdown, which explains the mismatch between the two viability assays. Collectively, the material preserved epidermal keratinocyte function relative to the blank control, yet reduced fibroblast metabolic activity, especially at 0.3%, and attenuated fibroblast proliferation and matrix synthesis. Thus, the 0.1% and 0.2% formulations benefit skin wound repair, while the 0.3% formulation poses potential risks for dermal regeneration relative to the blank control.

### 3.9. Scratch Wound Assay

**Figure 5:**
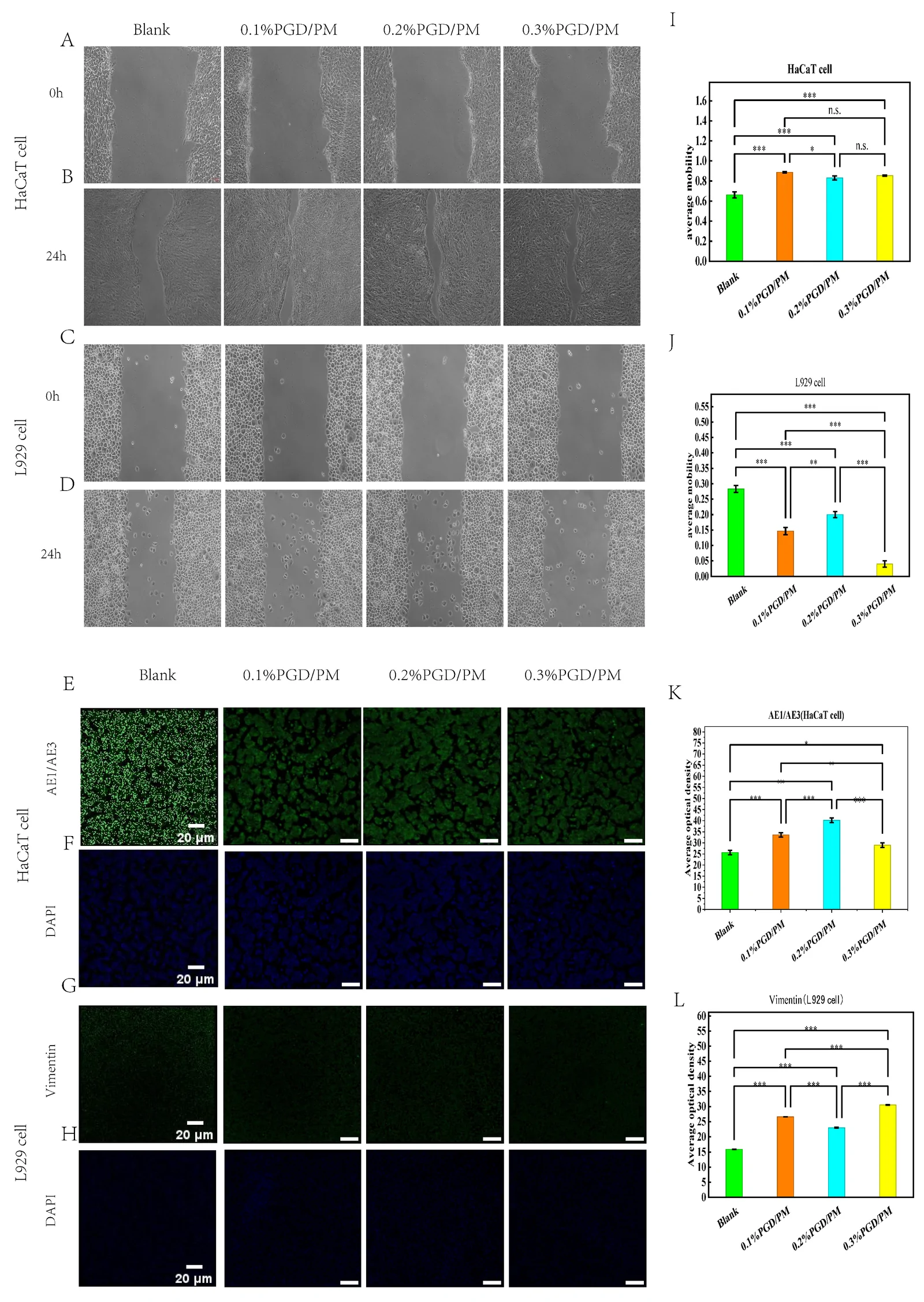
Cell migration and characteristic protein expression of HaCaT and L929 cells on nanofibre membranes. Representative scratch migration images of HaCaT (**A**,**B**) and L929 (**C**,**D**) cells at 0 h and 24 h, with quantitative migration rates (**I**,**J**). The cell viability of HaCaT cells was 66.33 ± 2.83% (blank), 88.67 ± 0.58% (0.1%), 83.00 ± 2.00% (0.2%), and 85.33 ± 0.58% (0.3%); L929 cell viability was 28.33 ± 1.15% (blank), 14.67 ± 1.15% (0.1%), 20.00 ± 1.00% (0.2%), and 4.00 ± 1.00% (0.3%). Immunofluorescence staining of AE1/AE3 in HaCaT (**E**,**F**) and vimentin in L929 (**G**,**H**) cells after 72 h culture, with quantitative average optical density analysis (**K**,**L**). Scale bar = 20 μm. * *p* < 0.05, ** *p* < 0.01, *** *p* < 0.001, and ns, no significance.

As shown in Figure 5A,B,I, the scratch wound assay showed that HaCaT cells cultured in the conventional medium exhibited a migration rate of 66.33 ± 2.83% at 0–24 h. Treatment with PGD/PM at all three concentrations significantly enhanced HaCaT cell migration: cells in the 0.1% group showed the highest migration rate (88.67 ± 0.58%), followed by the 0.3% (85.33 ± 0.58%) and 0.2% (83.00 ± 2.00%) groups, and there was no significant difference across these concentrations. In contrast, Figure 5C,D,J demonstrate that PGD/PM markedly inhibited L929 cell migration in a dose-dependent manner. Cells in the blank group showed a migration rate of 28.33 ± 1.15%, which decreased to 20.00 ± 1.00% (0.2%), 14.67 ± 1.15% (0.1%), and 4.00 ± 1.00% (0.3%) after PGD/PM treatment, with cells in the 0.2% group retaining the highest migratory capacity among the three treatment groups. Collectively, these results indicate that PGD/PM promotes keratinocyte migration while suppressing fibroblast migration. All three PGD/PM concentrations significantly enhanced the migration of HaCaT cells, thereby facilitating re-epithelialisation and accelerating wound closure. In contrast, PGD/PM exerted dose-dependent inhibition on L929 fibroblast migration, with the 0.3% concentration showing the strongest inhibitory effect. From the perspective of anti-scarring therapy, the suppression of fibroblast migration is beneficial because the MMC component effectively limits excessive fibroblast activation, infiltration, and hyperproliferation, which serves to reduce extracellular matrix overdeposition and prevent hypertrophic scar formation [36]. However, the inhibition of fibroblast migration also imposes potential disadvantages during the early stage of wound repair. Since dermal fibroblasts are the primary cells responsible for granulation tissue formation and extracellular matrix secretion, reduced fibroblast migration compromises early cellular recruitment and matrix deposition, which may delay initial wound reconstruction and weaken the structural foundation of regenerated skin [37]. Collectively, the findings show that PGD/PM exhibits dual regulatory effects on skin wound healing: it promotes rapid re-epithelialisation while moderately restricting fibroblast activity, achieving a balance between accelerated wound closure and scar inhibition, although high concentrations such as 0.3% may excessively suppress fibroblast function and hinder early dermal repair.

### 3.10. Immunofluorescence Staining

Staining with AE1/AE3, a broad-spectrum epithelial keratin marker, showed intense cytoplasmic staining in HaCaT cells, thereby confirming the stability of their epithelial phenotype. Quantitative immunofluorescence analysis (Figure 5E,F,K) further revealed sustained high levels of AE1/AE3 expression in the blank group and in all PGD/PM treatment groups (0.1%, 0.2%, 0.3%). Taken together, these findings indicate that epithelial-specific keratins remained robustly expressed on the nanofibre membranes under all tested conditions.

Vimentin [38,39,40], a type III intermediate filament protein and a canonical marker of mesenchymal cells, was used to assess whether the scaffolds could direct L929 cells towards anisotropic tissue formation. As illustrated in Figure 5G,H,L, vimentin expression was markedly upregulated in all PLLA/GEL/DMOG/PCL/MMC groups (0.1%, 0.2%, 0.3%) compared with the control group. This pattern suggests that the composite nanofibre membranes not only enhance mesenchymal marker expression but also provide excellent cellular compatibility, thereby supporting their suitability for skin tissue engineering.

Among the three experimental groups (0.1%, 0.2%, 0.3% PGD/PM), the 0.2% PGD/PM group exhibited balanced comprehensive performance. From the SEM images and fibre diameter statistics (Figure 2), the use of 0.2% PGD/PM formed uniform electrospun nanofibres with a suitable fibre diameter and porous structure, which was beneficial for cell attachment. The material possessed favourable wettability and mechanical properties to adapt to skin movement.

As shown in Figure 3, both the 0.2% and 0.3% groups achieved strong antibacterial activity against *S. aureus* and *E. coli*. Considering cytotoxicity (Figure 3), 0.3% PGD/PM slightly suppressed the viability of HaCaT and L929 cells, whereas 0.2% PGD/PM maintained high cell viability without obvious cytotoxicity. According to the scratch wound and immunofluorescence assays (Figure 4), 0.2% PGD/PM significantly promoted the migration of HaCaT keratinocytes and L929 fibroblasts and upregulated the expression of migration-related proteins. Although 0.3% PGD/PM showed comparable antibacterial effects, its higher concentration imposed potential cytotoxic risk. By contrast, 0.1% PGD/PM displayed insufficient bacteriostatic capacity. Collectively, as shown in Table 1, these findings show that 0.2% PGD/PM balances nanofibre morphology, antibacterial efficiency; and biocompatibility. Hence, it was selected for subsequent wound repair experiments.

**Table 1:** Physicochemical, antibacterial, and cellular biological properties of electrospun PGD/PM membranes with different PGD/PM concentrations.

Parameters	0.1% PGD/PM	0.2% PGD/PM	0.3% PGD/PM
Fibre diameter (μm)	0.261 ± 0.031	0.279 ± 0.028	0.291 ± 0.029
Water contact angle (°)	95.599	89.993	77.367
Antibacterial ratio against *S. aureus* (%)	>99	>99	>99
Antibacterial ratio against *E. coli* (%)	>99	>99	>99
HaCaT viability (live/dead staining, %)	94	96	93
L929 viability (live/dead staining, %)	92	94	93
HaCaT migration rate (%)	88.67 ± 0.58	83.00 ± 2.00	85.33 ± 0.58
L929 migration rate (%)	14.67 ± 1.15	20.00 ± 1.00	4.00 ± 1.00

### 3.11. Wound Healing

**Figure 6:**
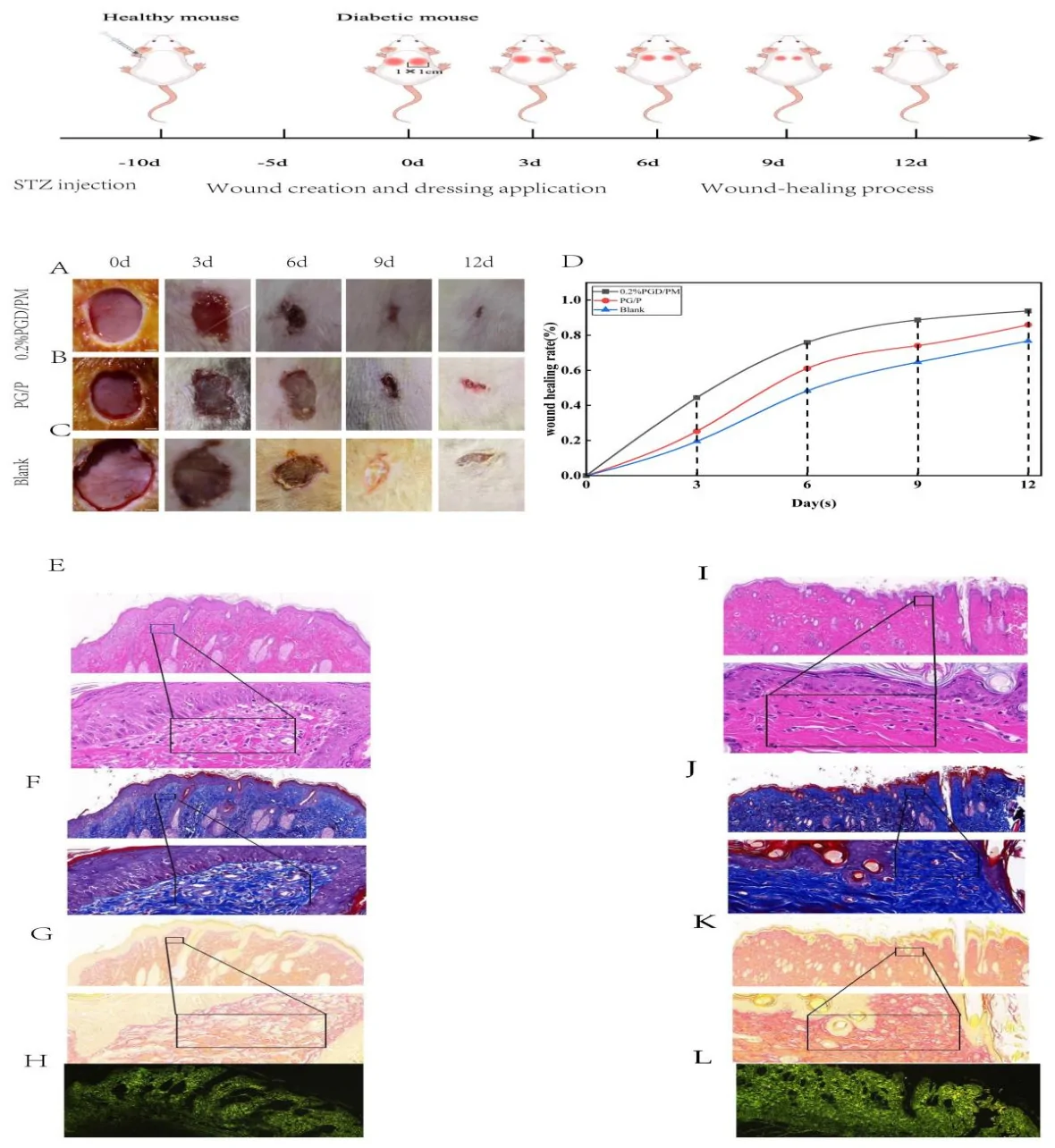
In vivo skin regeneration. Schematic timeline of the diabetic mouse wound model (**top**). Representative wound photographs of the 0.2%PGD/PM, PGP, and blank groups at 0, 3, 6, 9, and 12 days post-wounding (**A**–**C**). Quantitative wound healing rate of each group (**D**). Histological staining of regenerated skin tissue on day 12: H&E staining (**E**,**I**), Masson’s trichrome staining (**F**,**J**), Sirius Red staining under bright field (**G**,**K**), and polarised light microscopy (**H**,**L**); scale bar = 20 mm.

The wound healing cascade comprises haemostasis, cell proliferation and migration, inflammatory responses, and subsequent tissue regeneration [41,42,43]. As shown in Figure 6A–C, the therapeutic performance of PLLA/GEL/PCL, 0.2% PLLA/GEL/DMOG/PCL/MMC, and a blank control was assessed in a rat full-thickness skin excision defect model. Figure 6D summarises the temporal progression of wound closure, revealing that the wound healing rate increases progressively over the healing period. Notably, the DMOG/MMC-containing scaffold induced a significantly accelerated healing response relative to both the PLLA/GEL/PCL membrane and the blank control, whereas no significant difference was detected between the two scaffold-treated groups. As shown in Table 2, linear regression analysis revealed that the slope of the wound healing rate for the 0.2% PGD/PM group was 0.0784 day^−1^, which was higher than that of the PG/P (0.0701 day^−1^) and blank (0.0612 day^−1^) groups. The larger slope demonstrates the fastest daily increase in wound closure rate occurred in the 0.2% PGD/PM group. The porous nanofibrous structure provides abundant cell adhesion sites and a larger specific surface area [44], the latter of which offers a more sufficient interface for cell adhesion, proliferation, and nutrient/oxygen exchange, thereby promoting angiogenesis and tissue regeneration and accelerating wound healing [45]. The released DMOG effectively promotes keratinocyte migration and accelerates re-epithelialisation, while sustained MMC release suppresses bacterial infection and inhibits excessive fibroblast activation to reduce scar formation [19,20,31]. The PG/P group without loaded bioactive agents could only rely on the physical support of the nanofibre scaffold, thus showing a moderate healing slope. The blank group had the lowest slope because there was no biomaterial scaffold or therapeutic drug to facilitate wound healing. These findings, together with the degradation profiles, reveal that the 0.2% PLLA/GEL/DMOG/PCL/MMC scaffold degraded at a rate closely aligned with the intrinsic wound healing trajectory, thereby providing continuous structural and biological support throughout the critical 12-day repair window and preventing premature scaffold loss and wound re-exposure. By contrast, the PLLA/GEL/PCL group exhibited substantially delayed healing at later stages, attributable to the absence of synergistic functional components. This STZ-induced diabetic mice skin excisional wound model recapitulates several key features of clinical diabetic wounds, including sustained hyperglycaemia and impaired skin healing. However, this surgically created acute wound cannot fully mimic naturally occurring diabetic ulcers. The experimental wounds possessed a uniform shape and consistent depth, unlike irregular, long-standing clinical ulcers that are often accompanied by persistent infection, severe local vascular degeneration, and chronic inflammation. These differences constitute important limitations of the present animal model. These observations collectively indicate that the DMOG/MMC-loaded composite scaffold offers enhanced efficacy and a more favourable regenerative microenvironment for promoting cutaneous wound repair.

**Table 2:** Linear fitting parameters of wound healing rate over time in different groups.

Group	Fitted Equation	Slope (Day ^1^)	R^2^	Relative Healing Rate
0.2% PGD/PM	y = 0.0784x + 0.0421	0.0784	0.996	Fastest
PG/P	y = 0.0701x + 0.0186	0.0701	0.998	Intermediate
Blank	y = 0.0612x + 0.0037	0.0612	0.997	Slowest

^1^ Slope value calculated at Day 1.

### 3.12. Histopathological Staining

To evaluate cutaneous remodelling in mouse, HE and Masson staining were performed to examine the overall skin morphology at two weeks post-regeneration. As shown in Figure 6E,F,I,J the PLLA/GEL/PCL group displayed a discontinuous epithelial layer on the repaired skin surface, together with pronounced collagen deposition. By contrast, in the PLLA/GEL/DMOG/PCL/MMC (0.2%) group, the epithelial layer was regular and continuous, with abundant collagen deposition localised within the submucosal tissue. As shown in Figure 6G,H,K,L, Sirius red staining was used to characterise the formation and spatial distribution of collagen fibres in the skin; type III collagen appeared green, whereas type I collagen was visualised as red or yellow [46]. In hypertrophic scars, type III collagen is known to predominate [47]. Consistent with this, the PLLA/GEL/PCL group exhibited increased deposition of type III collagen and reduced deposition of type I collagen, as evidenced by the larger green regions. In contrast, the PLLA/GEL/DMOG/PCL/MMC (0.2%) group showed intense yellow staining indicative of type I collagen, closely resembling the collagen distribution observed in the control group, thereby suggesting that the newly formed collagen fibres in this group most closely approximated those of native skin tissue.

### 3.13. Immunohistochemical Staining Analysis

**Figure 7:**
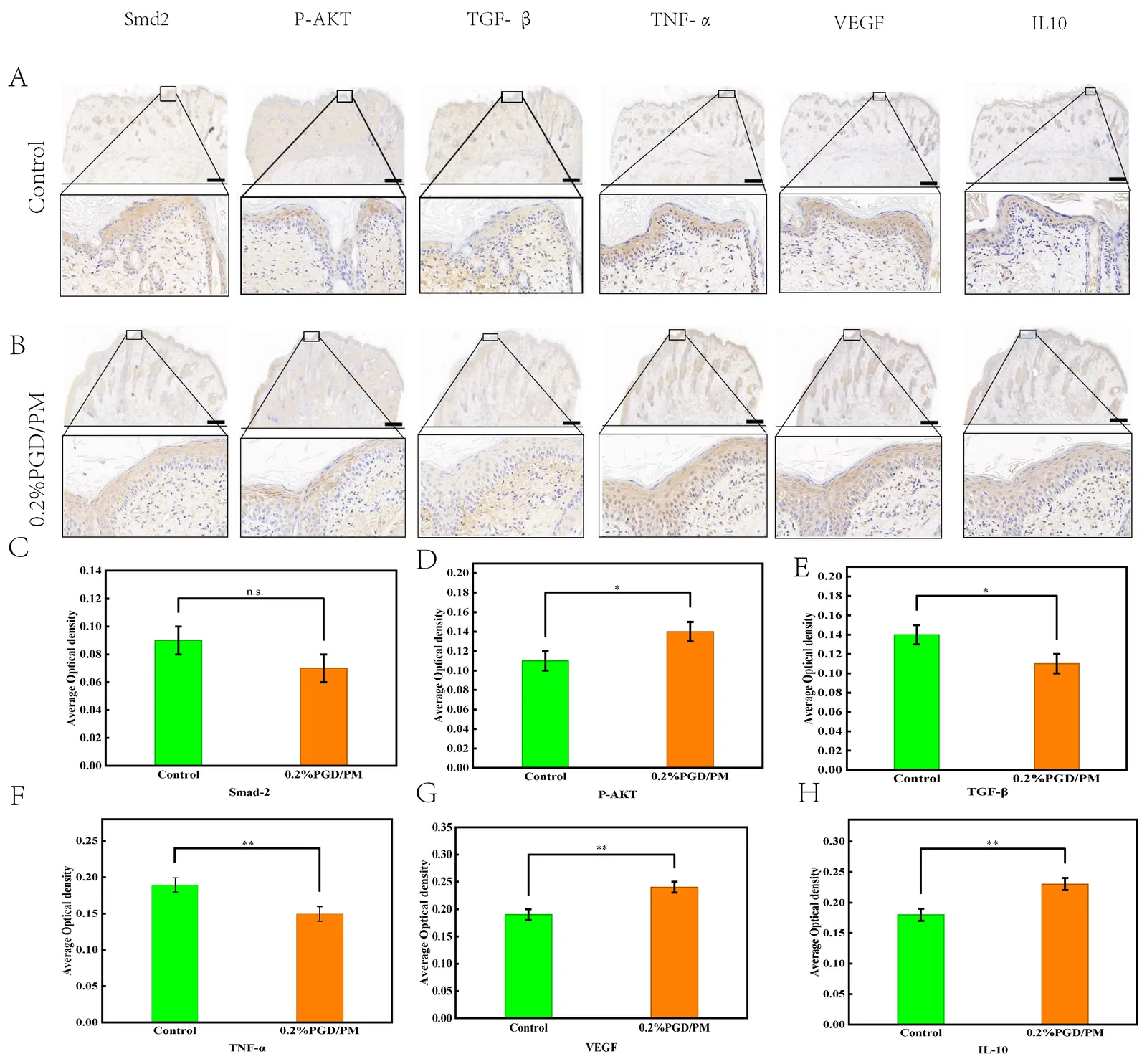
Immunohistochemical staining of regenerated skin tissues harvested at 2 weeks post-implantation. Representative micrographs of Smad2, P-AKT, TGF-β, TNF-α, VEGF, and IL-10 staining for the control group (**A**) and 0.2%PGD/PM group (**B**) (scale bar = 50 μm). Quantitative analysis of average optical density for Smad2 (**C**), P-AKT (**D**), TGF-β (**E**), TNF-α (**F**), VEGF (**G**), and IL-10 (**H**). * *p* < 0.05, ** *p* < 0.01, and ns, no significance.

As shown in Figure 6, immunohistochemical staining was performed to detect the expression levels of Smad2, P-AKT, TGF-β, TNF-α, VEGF, and IL-10 within wound tissue [48,49]. As shown in Figure 7C–H, the quantitative analysis of average optical density demonstrated that the 0.2% PGD/PM group exhibited significantly higher expression of pro-healing markers P-AKT and VEGF compared with the control group. The anti-inflammatory cytokine IL-10 was also markedly upregulated, whereas the pro-inflammatory factor TNF-α was distinctly downregulated. No significant difference in Smad-2 expression was observed between the two groups. TGF-β expression decreased moderately in the 0.2% PGD/PM group.

The elevated expression of p-AKT and VEGF suggests the potential activation of AKT-related signalling and upregulation of angiogenic-associated biomarkers in wound tissue, which may support nutrient delivery for granulation tissue formation and facilitate wound repair. The reduced TNF-α level together with the increased IL-10 level indicates that the 0.2% PGD/PM dressing mitigates local inflammatory responses, favouring the transition from the inflammatory phase to the proliferative phase of wound healing. The unchanged Smad2 level suggests that the Smad2-dependent signalling pathway is not significantly activated or suppressed under this treatment. Although TGF-β is moderately decreased, the available TGF-β may still support normal tissue repair. Collectively, these results indicate that the 0.2% PGD/PM dressing modulates the wound microenvironment by regulating inflammatory mediators and upregulating VEGF/p-AKT biomarkers, thereby promoting skin regeneration [50,51].

## 4. Conclusions

In summary, the 0.2% PGD/PM electrospun nanofibre membrane integrates favourable physicochemical and biological properties, making it a potential candidate for full-thickness skin wound repair. The appropriate hydrophilicity of this composite dressing enables efficient absorption of wound exudates and maintains a moist wound microenvironment, which prevents wound dehydration and scab formation and creates favourable conditions for tissue reconstruction. Moreover, the optimised surface wettability improves cell–material interfacial compatibility, which is conducive to stable cell adhesion, spreading, and continuous tissue–material interaction during the healing process. In terms of mechanical behaviour, this composite dressing exhibits moderate tensile strength and flexible deformability that match the mechanical characteristics of natural skin. Such mechanical compatibility allows the membrane to adapt to skin stretching and daily movements without detachment or fracture, thereby providing reliable physical protection for the wound bed against external mechanical irritation, secondary injury, and potential bacterial invasion.

In vitro cellular evaluations confirmed the good biocompatibility of the PGD/PM composite dressing. The electrospun nanofibre membrane showed negligible cytotoxicity toward HaCaT keratinocytes and L929 fibroblasts, which are essential cell types for skin regeneration. Its interconnected porous structure provides a three-dimensional microenvironment that supports cell adhesion, proliferation, and migration, thereby promoting the progression of skin tissue remodelling. Consistent with the in vitro results, in vivo immunohistochemical analysis demonstrated that the 0.2% PGD/PM dressing effectively modulated the wound microenvironment. The dressing upregulated the expression of pro-angiogenic-related proteins (p-AKT and VEGF) and anti-inflammatory cytokine (IL-10), while reducing the level of pro-inflammatory TNF-α. These regulatory effects alleviate excessive local inflammation, relieve inflammatory stress, and induce a pro-angiogenic response, which may facilitate the transition of wound healing from the inflammatory stage to the proliferative stage and accelerate skin repair.

Although immunohistochemical staining revealed upregulated expression of VEGF and P-AKT, suggesting that the 0.2% PGD/PM dressing may facilitate angiogenesis, several limitations remain regarding our assessment of angiogenic activity. In this study, representative angiogenic factors were only detected at the protein level via immunohistochemistry, without further quantitative validation using Western blot or qPCR at both protein and gene levels. Furthermore, we did not characterise microvessel density and the morphology of newly formed blood vessels via staining for endothelial-cell markers CD31 or CD34. In vitro functional angiogenesis assays, such as tube formation experiments, were also not performed to verify this composite dressing’s pro-angiogenic capacity. Accordingly, the current evidence for its pro-angiogenic effect remains indirect. Further studies integrating multiple molecular detection techniques and functional experiments are needed to comprehensively elucidate the angiogenic potential of this dressing. Regarding anti-fibrotic regulation, although a slight reduction in TGF-β expression was observed, the total Smad2 expression remained unchanged. Without detecting phosphorylated Smad2/3 and α-SMA expression and the ratio of collagen I/III, we cannot fully clarify the regulatory effect on the TGF-β/Smad signalling pathway or draw a robust conclusion regarding the inhibition of skin fibrosis. Therefore, the anti-fibrotic mechanism of the dual-drug scaffold remains preliminary. Multiple key histological and molecular fibrosis-related indicators were not examined, which is an acknowledged limitation of the current work. Collectively, the therapeutic effects of the PCL/PLLA/GEL-based composite scaffold should be interpreted cautiously based on the evidence obtained. The in vivo experiment consisted of three experimental groups: a blank control group, a PCL/PLLA/GEL base scaffold group, and an MMC/DMOG co-loaded scaffold group. This experimental setup only compared the therapeutic outcome of the dual-drug loaded membrane against the blank control and the unloaded base scaffold. It could not distinguish the independent biological contributions of MMC and DMOG, nor confirm whether any observed beneficial effect originates from MMC, DMOG, or their combined action. Additional in vivo groups carrying scaffolds loaded with MMC alone or DMOG alone were not included in this study; this lack of single-drug control groups is a notable limitation of our work.

Notwithstanding the above-mentioned drawbacks and experimental limitations, including the lack of in vivo long-term safety evaluation and insufficient exploration of deeper wound repair molecular mechanisms, the biomimetic electrospun composite dressing developed in this study still represents a highly promising candidate for full-thickness skin wound repair and scar management. Benefiting from its biomimetic nanofibrous structure, large specific surface area, favourable biocompatibility, balanced drug release behaviour, and dual functions of antibacterial infection prevention and scar inhibition, the optimised bilayer PLLA/GEL/DMOG/PCL/MMC membrane effectively overcomes the functional singularity and uncontrollable drug release of traditional wound dressings. It achieves coordinated regulation of early epithelial regeneration and later fibrotic remodelling, thereby supporting high-quality skin wound healing. Accordingly, further comprehensive and systematic investigations, including in vivo animal wound healing models, long-term biosafety detection, and deeper exploration of drug-mediated repair signalling pathways, are expected to validate this multifunctional wound dressing’s therapeutic efficacy and promote its clinical transformation and translational application.

## Data Availability

The original contributions presented in this study are included in the article. Further inquiries can be directed to the corresponding authors.

## Abbreviations

The following abbreviations are used in this manuscript:

PLLA	Poly(L-lactic acid)
GEL	Gelatine
DMOG	Dimethyloxalylglycine
PCL	Polycaprolactone
MMC	Mitomycin C
